# The value of life in English law: revered but not sacred?

**DOI:** 10.1111/lest.12131

**Published:** 2016-08-15

**Authors:** Rob Heywood, Alexandra Mullock

**Affiliations:** ^1^ UEA Law School; ^2^ University of Manchester

## Abstract

*Terms such as* sanctity *and* inviolability *have failed to provide a legally coherent or ethically sound principle upon which to determine the scope of the intrinsic value of life against extrinsic*, *quality‐of‐life considerations in a medical context*. *In their recent work*, *Margaret Brazier and Suzanne Ost introduce a new term*, reverence for life, *which they suggest may be more appropriate when attempting to navigate the murky waters of the meaning of life and the value that should be attached to it*. *They suggest that* reverence *should be utilised as an alternative that better reflects the nuances and the realities of the dilemma*. *This paper explores the existing difficulties before considering how the principle of* reverence *might provide a principled compromise over when the presumption in favour of preserving life should be rebutted*.

## Introduction

Understanding and interpreting the value of human life has troubled ethicists, theologians, philosophers and lawyers since the dawn of time. The extent to which life should be regarded as intrinsically valuable, weighed against the extrinsic value and quality‐of‐life considerations, is one of the most challenging and important legal questions. Unsurprisingly, the academic literature concerning this dilemma is voluminous. 1
See inter alia J Keown *The Law and Ethics of Medicine*: *Essays on the Inviolability of Human Life* (Oxford: Oxford University Press, 2012); R Dworkin *Life*'*s Dominion*: *an Argument about Abortion and Euthanasia* (New York: Alfred Knopf, 1993); J Harris The Value of Life (London: Routledge, 1985); G Williams The Sanctity of Life and the Criminal Law (London: Faber and Faber, 1957). Some prefer to use the language of sanctity of life, 2
See Williams, above n 1.
 whereas others suggest that this is misleading and instead refer to the inviolability of life. 3
See Keown, above n 1.
 In their recent work, Margaret Brazier and Suzanne Ost introduce a new term that they suggest may be more appropriate when attempting to navigate the murky waters of the meaning of life and the value that should be attached to it. 4


M Brazier and S Ost *Bioethics*, *Medicine and the Criminal Law Volume 3*: *Medicine and Bioethics in the Theatre of the Criminal Process* (Cambridge: Cambridge University Press, 2013).
 They suggest that the term *reverence for life* should be utilised as an alternative that better reflects the nuances and the realities of the dilemma. 5
Ibid, p 89.
 The language of reverence, it is argued, ‘might bridge the gaps between the different philosophical attitudes’ 6
Ibid.
 that infuse the debate concerning the acceptability of various end‐of‐life decisions. In this paper, we analyse evolving academic and judicial conceptions regarding the value of life in the context of dilemmas in end‐of‐life law. 7
‘End‐of‐life law’ encompasses both criminal and medical law principles. See J Coggon ‘Assisted dying and the context of debate: “medical law” versus “end‐of‐life law” (2010) 18 Med L Rev 541.10.1093/medlaw/fwq028PMC353719421098048



The delicate moral and ethical questions that underpin the meaning and value of life have taken centre stage in a number of recent high‐profile cases. 8
See, in particular, *M* *v* *N and Others* [2015] EWCOP 76; (2015) 18 CCL Rep 603; *R* (*Nicklinson and Another*) *v* *Ministry of Justice* , *R* (*AM* (*AP*)) *v* *DPP* [2014] UKSC 38; [2014] 3 WLR 200; *Aintree University Hospitals NHS Foundation Trust* *v* *James* [2013] UKSC 67; [2014] AC 591; *W* *v* *M* [2011] EWHC 2443 (Fam)10.1093/medlaw/fws01722798289; [2012] 1 WLR 1653; *R* (*Purdy*) *v* *DPP* [2009] UKHL 45; [2010] 1 AC 345; *Pretty* *v* *United Kingdom* (2346 / 02) [2002] 2 FLR 45; *Airedale NHS Trust* *v* *Bland* [1993] AC 789.
 It is clear from these cases that judges have sometimes struggled to separate the moral arguments from the legal ones. Attempting to explain and understand their reasoning by reference to a coherent judicial interpretation of the value that should be attached to human life has therefore become something of a challenge. Brazier and Ost suggest that adopting their language of reverence for life may be helpful in allowing us to rationalise the judgments that have touched on end‐of‐life. They suggest that cases should ‘rest on a strong presumption in favour of reverence for life’, 9
Brazier and Ost, above n 4, pp 90–91.
 but then further indicate that ‘the presumption is not and should not be irrebuttable’. 10
Ibid.



Drawing on the work of Brazier and Ost, this paper explores some of the conceptual difficulties inherent in assessing the value and importance that one should attach to human life. We assess how judges have come to understand and interpret the meaning and value of life by reference to a number of recent cases involving withdrawal of life‐sustaining treatment and physician‐assisted suicide (PAS). While these cases clearly raise different legal questions, resolving the profound disquiet over the value of life within all such decisions is a fundamental priority. Our analysis exposes some of the problems that have been left in the wake of contemporary case‐law, before identifying certain situations in which it may be possible to end life without offending certain core values and beliefs. We conclude by arguing that a more coherent and ethically consistent approach can and should be developed by means of the reverence principle. The principles of dignity and autonomy demand that a more consistent and principled approach is taken to identifying the circumstances that might rebut the presumption of preservation of life. While we do not have the space to examine in detail exactly when the presumption should be rebutted in every case, we suggest a starting point for a more productive and effective debate. Consequently, it is clear that the language of sanctity and/or inviolability presents a barrier to resolving the intractable tensions over this issue and thus, we argue, reverence for life is capable of capturing a workable compromise.

## Conceptual Difficulties with the Value and Importance of Human Life

One of the main problems when discussing the value of human life is the eclectic terminology that pervades the debate. The term ‘sanctity of life’ is commonly used in the literature and also in the case‐law. 11
In terms of the literature, see in particular Williams, above n 1. The sanctity of human life is discussed at length in the House of Lords’ decision in *Bland*, above n 8. More recently, see the judgment of Lord Neuberger in Nicklinson, above n 8, at [90]–[98].
 This, as Brazier and Ost point out, is a ‘misnomer as a description of how the criminal process in England does and should approach the value of lives’. 12
Brazier and Ost, above n 4, pp 83–84.
 Sanctity of life evokes strong religious connotations that do not sit squarely with modern conceptions of the role, aim and purpose of the criminal law. As Jackson states, the ‘idea that God alone should have the power to decide the moment of an individual's death’ 13


E Jackson and J Keown Debating Euthanasia (Oxford: Hart Publishing, 2012) p 37.
 is incongruous in a society that is no longer dominated by religious values and beliefs. The vernacular of sanctity has a tendency to give the impression that life should be preserved at all costs, a belief that is sometimes referred to as vitalism. 14
Keown, above n 1, p 4.
 In view of the sanctity of life being so closely aligned with religious beliefs, it has become possible for legal scholars to hone in on its weaknesses, identifying the fact that religion no longer dictates the contours of the criminal law. 15
Williams, above n 1.
 Thus, proponents of the intrinsic value of human life who deploy the language of sanctity to argue against allowing regulated assisted dying, for example, are immediately placed on the back foot.

Supporters of vitalism aside, very few scholars have tried to articulate an argument that supports preservation of life at all costs. Keown embraces the term ‘inviolability of life’, 16
Keown, above n 1, at 5–22.
 which for him removes the debate from the domain of religion and places it within the framework of the common law and human rights by recognising the intrinsic value of life itself. This allows Keown to construct a robust argument against allowing doctors to intentionally kill patients, which, he claims, is not based in religion, nor is inconsistent with fundamental principles of law. His criticism of those who elide the concepts of sanctity and inviolability is that they ignore key principles that are crucial to the sustainability of his argument. 17
Ibid, at 13–16, 332–335.



The key principle is that the inviolability of life prohibits intentional killing by act or omission. It follows that a doctor cannot intentionally shorten the life of his patient by undertaking a positive act to accelerate death, and equally is prohibited from withholding or withdrawing treatment, *with the intent to shorten life*. 18
Ibid, at 12.
 Accordingly, Brazier and Ost seek to distance themselves from the language of inviolability because they suggest it is ‘a more absolute command allowing no exception’. 19
Brazier and Ost, above n 4, p 89.
 Yet if one remains true to the inviolability ideology, this may not be an accurate interpretation. While the inviolability of life principle recognises no exceptions, it is perhaps misleading to say it is absolute in the sense that it holds that life should be preserved at all costs. The inviolability of life principle, as conventionally understood, permits the withholding and withdrawing of life‐prolonging treatment that is not worthwhile because it is futile or too burdensome for the patient. Thus, even though it would be wrong to withhold treatment because the patient's *life* was thought to be worthless, it would be acceptable to withhold treatment based on the fact that the *treatment itself* was deemed to be worthless, provided that the only intention of the doctor in withdrawing or withholding the treatment was to alleviate the patient's pain and suffering, with death being a foreseen consequence of that act. 20
Keown, above n 1, p 12.
 This position was described by David Price as a ‘sop’, 21


D Price ‘Fairly *Bland*: an alternative view of a supposed new “death ethic” and the BMA guidelines’ (2001) 21 Legal Stud 618 at 638.10.1111/j.1748-121x.2001.tb00183.x16184694
 in which Keown concedes some ground where a patient is being sustained in the most hopeless of situations, while at the same time enabling him to present what is, prima facie, an internally consistent argument that, in real terms, maintains an unduly restrictive approach to end‐of‐life decision making.

There are other more pragmatic concerns. First, the inviolability principle holds the doctrine of double effect in too high a regard, without considering the practical implications of the House of Lords’ decision in *R v Woollin*. 22


*R* *v* *Woollin* [1999] 1 AC 82.
 There are now some situations in which foresight of a virtually certain consequence *may* amount to intent and, as this question rests on the interpretation of a jury, any doctor seeking to rely on the double effect must surely be advised that she is traversing the most uncertain of legal terrains. Secondly, determining the question of futility is not straightforward. For every convincing argument in favour of suggesting that a treatment is in fact futile, a convincing counterargument can be raised. 23
See, amongst others, RK Mohindra ‘Medical futility: a conceptual model’ (2007) 33 J Med Ethics 71 1726419110.1136/jme.2006.016121PMC2598226; NS Jecker and RA Pearlman ‘Medical futility: who decides?’ (1992) 152 Arch Intern Med 1140 1599340; See also Sir Alan Ward's analysis in *Aintree University Hospitals NHS Foundation Trust* *v* *David James and Others* [2013] EWCA Civ 65 at [35].
 This allows the supporters of the inviolability principle to argue the case for justifiably sustaining a patient in a given situation. Finally, where the question converges on ‘treatment’, there is always room for disagreement as to what may actually amount to treatment. 24
For discussion, see Keown, above n 1, pp 330–332.
 Nonetheless, in theory at least, the inviolability principle is only absolute in the sense of the intrinsic value it affords to human life itself, but is not absolute in that it does not rule out withdrawal of treatment completely in certain types of cases.

The cases in which the inviolability principle would condone certain end‐of‐life decisions made by doctors are narrow and thus perhaps too restrictive. Some scholars therefore encourage the assessment of quality‐of‐life considerations when determining end‐of‐life questions. 25
See Jackson and Keown, above n 13; and also Harris, above n 1.



The contrasting view offered in resistance to Keown is that the value of life is self‐determined. Singer, for example, would suggest that a patient who is suffering from a terminal and/or degenerative illness should have the right to harbour her own view on the value of life and, as such, any right to life should not be enforced on her, mandating her to live against her will. 26


P Singer Practical Ethics (Cambridge: Cambridge University Press, 2nd edn, 1993) at 194–196.
 Harris holds a corresponding view and suggests ‘killing a person is only wrong when it deprives the person of something it values: there is no such wrong if the person no longer values life’. 27


J Harris ‘Euthanasia and the value of life’ in KeownJ (ed) *Euthanasia Examined*: *Ethical*, *Clinical and Legal Perspectives* (Cambridge: Cambridge University Press, 1997) pp 10, 20.
 Rachels frames a similar argument, albeit in a slightly different way. He suggests that life ought to be protected in a biographical sense, and not merely in a biological sense. Thus, ‘from the point of view of the living individual, there is nothing important about being alive except that it enables one to have a life. In the absence of a conscious life, it is of no consequence to the subject himself whether he lives or dies.’ 28


J Rachels *The End of Life*: *Euthanasia and Morality* (Oxford: Oxford University Press, 1986) p 26.
 Writers such as Singer, Harris and Rachels therefore believe that life is only of instrumental value, and that the worth of life ought to be self‐determined.

In framing their arguments in the above way, these scholars attempt to eschew the problems associated with any objective evaluation of quality of life. Where objective considerations creep into the equation, there is the risk that certain types of life will be significantly devalued. Thus, for example, there may be a tendency for someone looking through the lens of objectivity to suggest that the life of a severely disabled person has no worth, value or quality. Ergo, it ought to be more readily acceptable to prematurely end that life. The difficulty is that this view significantly undermines the position of disabled people in society, because they may be left with a feeling that their existence is worth less than that of an able‐bodied person. Thus, those opposed to any relaxation of the law in respect of PAS are quick to point out the dangers that naturally flow from calculations about quality of life made from an objective perspective.

Scholars such as Singer and Harris therefore remain resolute in their view that the value of life ought to be self‐determined, and that this is the strongest position from which to argue the case for legal reform. These perspectives are perhaps illustrated by reference to the widely reported plight of Dan James, a story that attracted widespread media attention in the build‐up to the House of Lords’ decision in *Purdy*. Dan James was involved in a rugby ground training accident and was unfortunately rendered tetraplegic as a result. After a number of unsuccessful suicide attempts, he eventually travelled to Dignitas in Switzerland, with the assistance of his parents, where he received help to end his life. After investigating the case, the DPP decided not to prosecute his parents, and a family friend, for the role they played in helping Dan James realise his wish to die. 29
For a discussion of the DPP's decision not to prosecute, see http://www.cps.gov.uk/news/articles/death_by_suicide_of_daniel_james/ (accessed 14 February 2016). See also *Purdy*, above n 8.



The temptation from some objective observers may well be to agree that Dan James was justified in making the decision that he did because there was no value in his life once he became permanently and irreversibly paralysed. However, some other objective observers may have an entirely opposite view. There may be those who suffer from a disability themselves – or, indeed, able‐bodied individuals – who would recognise worth in such a disabled life and who would argue that it remains a life worth living. An important concern for these individuals is that if it becomes acceptable to justifiably end a life, based solely on the grounds of disability, then a dangerous message is conveyed to those with a disability. Moreover, occurrences of assisted suicide might escalate and perhaps spiral out of control.

Maintaining that the value of life ought to be self‐determined avoids some of the difficulties encountered by objective generalisations, which may devalue certain types of life. Thus, it could be argued that assessing the value of Dan James’ life, through the lens of Dan James himself, is the correct way in which to determine the worth of life. If asked, before his accident, Dan James undoubtedly would have testified to the fact that his life had quality, and that for him it was well worth continuing to live. However, after his tragic accident, viewed from his own perspective, this changed. His existence no longer had sufficient quality and value, *to him*. As such, he was justifiably entitled to make a decision to die, and should have been legally allowed to receive help.

At this stage, a number of issues become visible. First, whichever perspective is adopted, there is no one formula for reaching a view on the value of life and, 30
For discussion, see J De Haan ‘The ethics of euthanasia: advocates’ perspectives’ (2002) 16 Bioethics 154 at 171 1208315610.1111/1467-8519.00276; R Huxtable and M Moller ‘“Setting a principled boundary”? euthanasia as a response to “life fatigue”’ (2007) 21 Bioethics 117.1784548310.1111/j.1467-8519.2007.00535.x
 as Huxtable has incisively observed, there is no escaping the fact that in some situations in reality what is being claimed is ‘that a patient enduring a poor quality existence might be better off dead’. 31


R Huxtable *Euthanasia*, *Ethics and the Law*: *from Conflict to Compromise* (London: Routledge Cavendish, 2007) p 15.
 This, of course, is anathema to scholars such as Keown and thus a pattern emerges in which it becomes evident that views pertaining to the value of life, and the desirability of being able to choose the manner and timing of one's death, and in some instances to receive assistance in doing so, are polarised.

Secondly, different issues are at stake in different types of cases. On one view, arguments about reverence and a rebuttable presumption in favour of life seem perhaps more plausible where the patient lacks capacity to express a view about the worth of their own life, as opposed to a situation in which a patient is able to express *their* own views about the importance they attach to *their* life. In the latter, one may intuitively think that conceptions of autonomy ought to be afforded greater priority and can do all the work, but this offers too simplistic a view of the relationship between the law per se and autonomy, and we penetrate deeper into this point as our analysis progresses. At present, though, it suffices to say that we acknowledge that different issues are in play where the patient lacks volition. Nevertheless, we also suggest later in the piece that, while our arguments may be stronger in PVS/MCS cases, the concept of reverence also has a role to play in situations where active assistance is sought.

Before we move on to consider how Brazier and Ost's use of ‘reverence’ may usefully adopt a middle ground between two extreme positions, it is first necessary to explore the origins of the concept itself.

## Reverence for Life: Exploring the Term

Brazier and Ost do not claim ownership rights of the term ‘reverence for life’. Albert Schweitzer introduced the phrase itself into modern ethical discourse. 32


A Schweitzer The Teaching of Reverence for Life (London: Peter Owen, 1966).
 His notion of reverence for life was born out of a quest ‘to find the elementary and universal concept of the ethical’, which he ‘had not discovered in any philosophy’. 33


A Schweitzer *Out of My Life and Thought*: *an Autobiography*, tr AB Lemke (New York: Henry Holt, 1990) pp 154–155.
 The ethic itself contains a multifaceted set of tenets, yet a central theme is that the principle of reverence for life recognises ‘as good only the preserving and benefiting of life: any injury to, and destruction of, life, unless it is imposed upon us by fate, is regarded as evil’. 34
Schweitzer, above n 32, p 31.
 Thus, it is argued that ‘in every case we must decide ourselves to what extent we may remain ethical and to what extent we will have to bow to the necessity of harming and destroying a life, and thereby incurring the guilt of such actions’. 35
Ibid.



On one level, then, it may seem perplexing that Brazier and Ost have sought to reintroduce a term into the value of life debate that has hitherto been understood as not having ‘a large stock of compromises’ and is premised on a gripping ‘desire to preserve and benefit life’. 36
Ibid.
 While it is true that there are elements of Schweitzer's philosophy that acknowledge the importance of compassion and the justified ending of a life out of necessity in certain situations, his acceptance that some ethical activity may entail killing extends only to non‐human life. Indeed, a criticism of his thesis is that his notion of compassionate ending of life does not extend to humans 37
See AP Barsam *Reverence for Life*: *Albert Schweitzer*'*s Great Contribution to Ethical Thought* (Oxford: Oxford University Press, 2008) pp 36–37.
, which is encapsulated in the two statements that ‘reverence for life orders us not to take even the … agonizing life of man’ and ‘with a suffering person … I ought not to shorten his life even by an hour’. 38


A Schweitzer *A Place for Revelation*: *Sermons on Reverence for Life*, ed StregeM and StiehmL, tr DL Holland (London: Macmillan, 1988) p 37.
 It follows that some considered reflection is required as to how, if indeed at all, the concept of reverence for life, as used by Brazier and Ost, can be differentiated from the traditional understanding associated with Schweitzer's philosophy.

Brazier and Ost, while acknowledging the origins of reverence for life as being grounded in the work of Schweitzer, submit that they do not intend to ‘use the phrase to reflect in full Schweitzer's central ethic’. 39
Brazier and Ost, above n 4, p 89.
 However, while they do state that they share his emphasis on the intrinsic value of all life, they do not elaborate to any great extent on the major differences in their usage of the term. 40
Ibid. The authors simply state that it will become ‘apparent’ that they do not intend to use the phrase in the same way as Schweitzer.
 It is crucial to articulate these differences in order that Brazier and Ost do not get accused of merely reinventing the wheel, or that the term they are relying on does not capture accurately the message that they are attempting to convey.

A key aim of Brazier and Ost in employing reverence is to remove the debate from the domain of religion, at least to an extent. There may be a degree of scepticism as to whether or not this objective is actually met. Reverence, it is argued, ‘lacks the connotations of “holiness” inherent in sanctity, and the rather exclusive connections that link sanctity to Christianity’. 41
Brazier and Ost, above n 4, p 90.
 This may well be true, but the author's themselves concede that reverence also carries with it ‘religious overtones’. 42
Ibid.
 Moreover, if one looks to the original philosophy of reverence, while there is some debate about it, it has been pointed out by one prominent scholar that Schweitzer's reverence for life has a ‘religious character’ and contains the ‘surmising and longings of all deep religiousness’. 43
See Barsam, above n 37, p 30. See also Schweitzer, above n 33, p 237.
 Does the subtle linguistic variance of reverence really alter anything, then, by departing from the holiness of sanctity, and the more restrictive position of inviolability, or is it just being deployed as a smokescreen by Brazier and Ost, serving as little more than an agreeable term used to mediate between their own differences of opinion concerning the law in regard to end‐of‐life decision making? We agree that the manner in which Brazier and Ost propose to use reverence is inherently different to the classic philosophy of Schweitzer and we also agree that it could actually make a practical difference in cases that touch on end‐of‐life issues. However, in order to make that difference more pronounced, it is perhaps more appropriate to concentrate on the substance behind the language of Brazier and Ost's reverence in addition to focusing on the language itself.

Brazier and Ost suggest that in adopting the term ‘reverence for life’, their aim is not to answer all the questions. In actual fact, once again, this attitude is not dissimilar from at least one reading of Schweitzer's original philosophy, which suggests that reverence is not meant to establish specific rules for each possible circumstance, but is more concerned with creating a general attitude of universal reverence. 44
See Barsam, ibid, p 40.
 To this end, both classical and contemporary uses of reverence bear at least some resemblance. Nevertheless, the two ideas do diverge. Whereas Schweitzer was concerned with developing reverence as a universal ethic, Brazier and Ost are more concerned with ‘offering a framework in which the role of the criminal process can be located’. 45
Brazier and Ost, above n 4, p 90.
 Remaining cognisant of the fact that reverence is being used here only as a framework is vital, as it allows us to consider carefully the entwined relationship between the value of life, the law and autonomy. It becomes especially pertinent to the argument that we construct later in this piece that reverence has a role to play not only in withdrawal of treatment cases, but also in active assistance cases. A relevant question at this stage, though, is how Brazier and Ost envisage the operation of this framework, as this marks out the most obvious departure from Schweitzer's philosophy and where the greatest potential for impact could be achieved.

Whereas both Schweitzer, and Brazier and Ost, share the sentiment of the intrinsic value of all life, Brazier and Ost's point of departure rests on the notion of a rebuttable presumption. In regard to human life, Schweitzer would seemingly reject any notion of being able to rebut the intrinsic value of life and for him it would be unethical, and abhorrent, to ever foreshorten human life, no matter what the circumstances. Brazier and Ost's proposal, however, focuses on a presumption in favour of reverence for life, by which they recognise the intrinsic value of human life itself. Yet, they concede that this presumption is capable of being rebutted, and indeed should be in appropriate circumstances. 46
Ibid, p 91.
 Their use of reverence for life would thus permit the ending of human life in certain situations, if the facts of a given scenario were so compelling so as to provide convincing evidence that any emphasis placed on the intrinsic value of life was capable of being outweighed by competing factors that ought to allow the law some room to manoeuvre away from the absolute position that life needs to be preserved at all costs. Used in this way, reverence contains the hallmarks of a type of compromise, and so a picture begins to emerge in which the midpoint of reverence could have the potential to impact upon the outcome of certain cases. The systematised use of the term could develop a more principled approach in which judges’ minds are more focused upon the delicate balance that needs to be struck when questions are at stake concerning the end of life. It may cause them to become more sensitised to the issues by, on the one hand, not losing sight of the importance of the intrinsic value of life and, on the other, developing an acute awareness that it should not be afforded absolute priority in every given case and that certain factors are capable of overriding it.

It is not the first time that the idea of compromise per se has been posited in the field of end‐of‐life decision making, 47
For an alternative and interesting discussion, see R Huxtable *Law*, *Ethics and Compromise at the Limits of Life*: *to Treat or Not to Treat*? (London: Routledge, 2013).
 but the aspect of Brazier and Ost's work that marks it out as being especially interesting is the manner in which they feel compromise could be achieved and the evidence that would be required in order to rebut the presumption.

In order to assess how the term ‘reverence for life’ may have been effectively relied upon in the recent case‐law concerning the meaning and value of human life, it is first helpful to consider why it may be more appropriate, and in turn effective, to consider things from the perspective of a rebuttable presumption.

## Accommodation, Exception or Rebuttable Presumption?

Those who remain committed to the inviolability of life principle have always been critical of the House of Lords’ decision to sanction the withdrawal of life‐sustaining treatment in *Bland*. 48

*Bland*, above n 8. For a general critique, see Keown, above n 1.
 Throughout its protracted lifespan, judges in the various courts in which the case was heard sought to reassure us that their decision did not cast off the intrinsic value that the law has always placed on life. For example, Hoffmann LJ (as he then was) in the Court of Appeal declared:

In my view the choice the law makers must reassure people that the courts do have full respect for life, but they do not pursue the principle to the point at which it has become almost empty of any real content and when it involves the sacrifice of other important values such as human dignity and freedom of choice. 49


*Airedale NHS Trust* *v* *Bland* (1992) WL 896030; (1992) 142 NLJ 1755, per Hoffmann LJ (as he then was).

The criticism of the decision, it is claimed, is based on the reasoning and not the outcome. Keown suggests that their Lordships focused their minds on the wrong question when they considered whether the patient's *life* itself was worthwhile, rather than assessing the question of whether or not the *treatment* being provided was worthwhile. He argues that the correct questions that should be asked in withdrawal cases are: ‘is tube‐feeding “treatment” and, if so, is it worthwhile?’ 50
Keown, above n 1, p 340.
 However, even if these ‘correct’ questions had been asked in *Bland*, there is still a convincing argument that the treatment should still have been withdrawn. Yet, would the supporters of the inviolability of life ever truly admit this and accept *Bland* for the common‐sense and humane decision that it was? One suspects not. The argument would undoubtedly have been put forward that the artificial nutrition and hydration (ANH) was not treatment but rather ‘basic care’ that can never be withdrawn, or if ANH *is* treatment, that the threshold for futility ought to be incredibly high and was therefore not met.

To suggest that ANH is not medical treatment is a particularly weak argument that has been dismissed judicially, 51

*Bland*, above n 8.
 yet, for the interpretational reasons noted earlier, the futility question is delicately poised and difficult to deal with. The issue becomes even more vexed when considering futility from the perspective of benefits versus burdens. 52
This approach is actually advocated by Keown, above n 1.
 In many respects, the assessment of the benefits versus burdens of treatment is actually a useful starting point in the context of assessing futility, but some would argue the calculation should not be confined solely to treatment per se, and so the assessment of benefits and burdens will not always be as clear‐cut as it was in *Bland*.

In recent times, the courts have been faced with a number of challenging scenarios. Where the patient is in a minimally conscious state (MCS), the assessment of futility demands a more detailed forensic examination from a judge. The fact that the patient is receiving some benefit from the treatment is much easier to identify than in, say, a patient in a persistent vegetative state (PVS), but whether or not the burdens of the treatment outweigh the benefits is a different matter. In cases such as *W v M* and *Aintree*, 53


*W* *v* *M* ; *Aintree*, above n 8.
 explored in more detail below, judges have adopted varied approaches to the question of futility, with some placing emphasis on only the benefits of treatment as a reason for preserving life, and others implementing a more expansive balancing exercise yielding a rather different result.

To categorise the withdrawal of treatment cases as exceptions to the inviolability of life principle may be misleading. Its supporters would simply suggest that, if correctly reasoned, withdrawal of life‐sustaining treatment where the treatment itself is deemed futile is not ruled out and can thus be accommodated within the principle. Judges have nonetheless been slow to explain and justify the decisions in which they have permitted withdrawal of treatment by reference to acting within the margin of acceptability recognised by the classic inviolability principle. Equally, the situations in which they could actually do so, if they thought it appropriate, are limited to withdrawal type cases and, for this reason, it may be necessary to identify actual exceptions to the fundamental belief that recognises the intrinsic value of life.

As noted above, inclusive of, but not limited to, withdrawal of treatment cases, there are those at the opposite end of the spectrum to the inviolability principle who suggest it is appropriate to consider factors pertaining to the patient's quality of life. 54
See discussion above at pp 6–8. In particular, see L Doyal ‘Dignity in dying should include the legalisation of non‐voluntary euthanasia’ (2006) 1 Clin Ethics 65 ; Singer, above n 26.
 From this reasoning a strong argument emerges in favour of greater flexibility within the law that not only allows passive withdrawal of treatment, but also the intentional and active shortening of a patient's life in certain circumstances. This approach is still problematic for some though, as to distance oneself from recognising *any* intrinsic value to human life is too radical a step. 55
Indeed, this would seem to be a key reason as to why Brazier and Ost have sought to develop their reverence for life argument.



In view of this, Brazier and Ost's suggestion of a rebuttable presumption in favour of the intrinsic value of life, in the form of reverence for life, could act as a happy medium. 56
Brazier and Ost, above n 4, at 90–91.
 Whether or not one chooses to view it as an advantage or disadvantage, reverence would certainly be wider in scope than the permissible withdrawal scenarios accommodated within the inviolability principle; it could conceivably be extended to permit active intervention, encompassing cases such as *Pretty*, *Purdy* and, more recently, *Nicklinson*. 57

*Pretty*, *Purdy* and *Nicklinson*, above n 8.
 Tensions will naturally surround the concept of a ‘rebuttable presumption’. Some thought would need to be given to the precise nature and type of evidence that may be required in order to rebut the presumption in favour of reverence for life. There would need to be convincing evidence that the plight of the patient did fall within one of those ‘hard’ cases in which there was a solid justification for permitting a patient's life to be shortened, whether by passive omission or active assistance, and regardless of whether it was accompanied by a direct intention to shorten life. Identifying those ‘hard’ cases is where the difficulty will lie, but certainly the recent decisions in *W v M*, *Aintree* and *Nicklinson*, 58


*W* *v* *M* ; *Aintree*; *Nicklinson*, above n 8.
 to which we now turn our attention, are strong candidates for falling within this category. The legal determinations required in *Aintree*, for example, compared to *Nicklinson*, 59
Ibid.
 are, of course, palpably distinct. Yet within all these cases the question over the extent to which the law can, and *should*, allow medical interventions (acts or omissions) that intentionally cause death, is a fundamental concern. For this reason, we suggest that the reverence principle is capable of enhancing the resolution of all such cases that involve medical matters of life and death.

## Recent Judicial Interpretations of the Value of Life

The following cases compelled members of the judiciary to consider how the ‘sanctity’ principle should be weighed against other factors, principles and laws. We assess how the presumption that human life has intrinsic value was regarded in the context, and against the conflicting issues, of each case.

- ***W v M***

In this case, Baker J prioritised the sanctity of life in the face of compelling evidence that M, who was minimally conscious, would not wish to be kept alive in such a condition. We have both (separately) argued that this narrow evaluation gave too much weight to the intrinsic value of M's life in the best interests balancing exercise, 60
See R Heywood ‘Withdrawal of treatment from minimally conscious patients’ (2012) 7(1) Clin Ethics 10–16 ; A Mullock ‘Deciding the fate of a minimally conscious patient: an unsatisfactory balancing act? (2012) 20(3) Med L Rev 460–469 10.1093/medlaw/fws01722798289; A Mullock ‘Best interests and the sanctity of life after *W v M*
’ (2013) 39(9) J Med Ethics 553–554.2317290010.1136/medethics-2012-100907
 while giving too little weight to her past wishes and feelings, 61
Section 4(6) of the Mental Capacity Act 2005 requires the decision maker to ‘consider, as far as reasonably ascertainable, the person's past and present wishes and feelings … and the beliefs and values that would be likely to influence his decision …’
 the views of her family 62
Mental Capacity Act 2005 s 4(7). For an interesting discussion, see S Halliday , C Kitzinger and J Kitzinger ‘Law in everyday life and death: a socio‐legal study of chronic disorders of consciousness’ (2015) 35(1) Legal Stud 55–74.10.1111/lest.12042PMC445022026041944
 and also the grim reality of life at the lower limits of MCS. In the absence of a legally binding advance decision to refuse life‐sustaining treatment (AD), which would have rebutted the presumption of preserving life, Baker J felt that the evidence of M's sister and partner regarding M's wishes (that she would not wish to be kept alive in a state of total dependency), was insufficient to justify withdrawing ANH. The main reason given was that in contrast to a person in a vegetative state, a minimally conscious person is capable of experiencing life, whatever that might mean for *that* person. 63
The evidence produced painted a very uncertain picture about M's quality of life, including evidence of suffering, distress and some contentment. Some have questioned the assumption that something is better than nothing in this context. See eg S Ashwal and R Cranford ‘The minimally conscious state in children’ (2002) 9 Seminars in Pediatric Neurology 19.1193112210.1053/spen.2002.30334
 However, Baker J indicated that the presumption in favour of preserving life should only (definitely) continue if M's clinical condition remained stable. He intimated that if future, even trivial, infection threatens M's life, it might be appropriate not to treat her. This tells us that the presumption can be highly opportunistic and will often depend upon the means of achieving death, a phenomenon that has been considered by Kitzinger and Kitzinger, regarding the ‘window of opportunity’ for allowing death in. 64


C Kitzinger and J Kitzinger ‘The “window of opportunity” for death after severe brain injury: family experiences’ (2013) 35 Sociol Health & Illness 1095.10.1111/1467-9566.1202023278317
 Accordingly, for Baker J, the appropriate window for rebutting the presumption for preserving M's life in the absence of a binding AD would be if an infection struck and the treatment to be withheld is not ANH, or at least not only ANH.

***Aintree***

Again, this case concerned a best interests evaluation in order to determine whether treatment should be withheld for a patient in MCS. 65
The treatment in question was not ANH but invasive support for circulatory problems, renal replacement therapy and CPR (in the event of cardiac arrest).
 Unlike M, however, the evidence suggested that the patient, David James, initially experienced a greater degree of consciousness before rapidly deteriorating to the point at which death seemed imminent. At the point at which he had a sufficient degree of response and awareness, the evidence indicated that he might have expressed a wish for treatment to continue in order to prolong his life. With or without treatment, however, the clinical evidence indicated that Mr James was approaching the end of life, and indeed he died before the Supreme Court considered his case. Considering the decision of Jackson J in the Court of Protection, (broadly) in favour of continuing treatment, 66

*Aintree* [2012] EWHC 3524 COP.
 and the subsequent Court of Appeal decision that overturned Jackson J's decision, 67

*Aintree* [2013] EWCA Civ 65.
 the Supreme Court sat as a panel of five, 68
Lord Neuberger, Lady Hale, Lord Clarke, Lord Carnwath and Lord Hughes.
 reaching a unanimous decision delivered by Lady Hale.

Confirming that the ‘strong presumption that it is in a person's best interests to stay alive’ 69

*Aintree*, above n 8, at [35].
 is not an absolute position, Lady Hale scrutinised the approach to the best interests test in order to establish a more coherent principle regarding its possible rebuttal. Lady Hale agreed with the Court of Appeal's decision that it was not in Mr James’ best interests to have the treatments because by the time the case had reached them his health had significantly deteriorated. Yet, she held that their reasoning and approach to the best interests test had been wrong. Rather, Jackson J had been correct in his broad approach, which considered the question of futility from a subjective rather than primarily objective perspective, taking greater account of the patient's apparent wishes and what he might regard as a worthwhile treatment. Thus, a treatment that delivers ‘some benefit’ to the patient, even if it does not affect the underlying disease, might be regarded as worthwhile despite its limited clinical value. The nuances of the case concerning the particular treatments, however, led Lady Hale to the conclusion that Jackson J had overlooked some aspects of the consequences and burdens of some of the treatments. Moreover, notwithstanding the subjective influence upon the question of futility, objective clinical appraisal of the burdens of each treatment remained necessary and that as patients, ‘[W]e cannot always have what we want’. 70
Ibid, at [45]. This supports the decision in *R* (*Burke*) *v* *General Medical Council* [2005] EWCA Civ 1003 CA.



Whether or not the above message has been received and wholly understood is perhaps debatable, particularly in light of contemporary developments. English law does not endorse the substituted judgement approach, in which the decision maker attempts to step into the shoes of the patient. 71
Substituted judgment is employed in some US jurisdictions. See *In re Quinlan* (1976) 355 A.2d 647; *Cruzan* *v* *Director Missouri Department of Health* (1990) 110 S Ct 2841 (USA Supreme Court).12041283
 Best interests, at least in theory, remains an objective question, with some very clear subjective elements that need to be considered within that overall assessment. Nevertheless, the recent case of *M v N*, 72


*M* *v* *N* , above n 8.
 which represents the first time in which a judge in the Court of Protection has countenanced the withdrawal of ANH treatment from a patient in a MCS, seems to indicate that the objective component to the best interests assessment may be withering on the vine.

Hayden J, in concluding that the treatment could be withdrawn, seemed to justify his decision *solely* by reference to what he thought the patient would have wanted to happen. In suggesting that there ‘was no prospect of her achieving a life that **she** would consider to be meaningful, worthwhile and dignified’ 73
Per Hayden J in *M* *v* *N* , above n 8, at [74].
 (emphasis of Hayden J) and that it would be ‘disrespectful to preserve her further in a manner I think **she** would regard as grotesque’ 74
Per Hayden J in *M* *v* *N* , above n 8, at [75].
 (emphasis of Hayden J), there is clear evidence that the subjective views of the patient were prioritised. In effect, his judgment was advocating a substituted judgement approach in everything but name, at the expense of giving serious consideration to any objective factors that may well have been relevant and could in fact have been pulling in the opposite direction. 75
For another recent case in which significant emphasis was placed on the subjective position of the patient, see *Wye Valley NHS Trust* *v* *B* [2015] EWCOP 60; [2015] COPLR 843.



While it is fair to say that we welcome the outcome in the case, to transform the best interests assessment into a *wholly* subjective assessment is potentially problematic. First, the substituted judgement approach is not always autonomy enhancing. This is because in attempting to view things through the eyes of the patient, a judge is still effectively reaching *her* own conclusions about what *she* thinks the patient would want to happen. These are the judge's views, not the patient's. 76
This is especially pertinent in the light of the UN Convention for the Rights of People with Disability (UNCRPD), Art 12. This provision seeks to promote a ‘supported decision‐making' scheme for those who lack capacity, which creates a tension if the patient seems to prefer a course of action that is not in his medical best interests. Also, if the patient is unable to communicate or indicate any preference, it would appear to be effectively impossible to achieve ‘supported decision‐making’.
 An inescapable consequence of this is that in certain situations objective considerations may creep into a judge's reasoning and influence what she thinks about what the patient would want to happen. Attempting to disguise this, via recourse to a notion of substituted judgement, is dangerous and it is perhaps better that judges make overt reference to any objective considerations within a more rounded balancing exercise.

Secondly, attempting to view things entirely from the patient's perspective overlooks the fact that in some cases there may well be objective evidence that is more compelling and persuasive, and that could point in either direction if correctly reasoned. It is with these above dangers in mind that reverence for life once again becomes useful, as it would not seek to completely privilege any one approach and would encourage a more holistic and balanced best interests assessment that takes all factors, both subjective and objective, into account when determining whether there was enough evidence in a given case to rebut the presumption in favour of life. Moreover, as one of us as argued in more detail, a more expansive evaluation that considers the wider social issues together with a more nuanced approach to questions of futility and intolerability is necessary to avoid an overly medical, paternalistic approach. 77


R Heywood ‘Moving on from *Bland*: the evolution of the law and minimally conscious patients’ (2014) 22(4) Med L Rev 548–571.10.1093/medlaw/fwu00324618294



Reviewing other factually similar cases coming before the Court of Protection since *Aintree*, the signs do indicate that judges are approaching the balancing exercise in a more holistic manner. For example, in *United Lincolnshire NHS Hospitals v N*, 78
[2014] EWCOP 16.
 the court decided not to reinstate ANH for a woman whose feeding tube had become dislodged and who was physically resistant to attempts to reinsert it. N was in a very similar condition to M and, according to her next of kin, she would have found it an affront to her dignity to be subjected to continued interventions to maintain her life in that condition. 79
Ibid, at [29]–[33].
 Unlike M, however, N's unequivocally negative physical response to attempts to reinstate feeding seemed to indicate physical opposition to treatment, as well as indicating that further attempts might be clinically unsuccessful and thus futile. Accordingly, the court had more than N's past expressions of preference to weigh in the balancing exercise.

Also interesting is a case involving a minimally conscious man who was clinically stable and whose partner and close friends gave evidence that clearly indicated he would wish ANH to be withdrawn. 80


*Sheffield Teaching Hospital NHS Trust* *v* *TH and TR* [2014] EWCOP 4.
 Mr Justice Hayden was critical of the Official Solicitor's apparent reluctance to accept a more holistic approach to determining best interests:

I must record that the Official Solicitor's lawyers appear not to share my analysis of the cogency and strength of TH's wishes regarding his treatment. I confess that I have found this surprising. If I may say so, they have not absorbed the full force of Lady Hale's judgment in *Aintree* and the emphasis placed on a ‘holistic’ evaluation when assessing both ‘wishes and feelings’ and ‘best interests’. 81
Ibid, at [55].

Ultimately, no decision was made at the time because the case was adjourned pending further medical evidence, yet we can detect in Hayden J's words a clear willingness to fully respect the patient's autonomy and dignity, a position that he later concretely reinforced in *M v N*.

In relation to assisted dying, the dilemma over preserving life is in some respects less troubled when we know unquestionably that a person wants to die, provided, of course, that they are able (and willing) to act alone. Here it could be reasonably argued that the autonomous choice of the patient is all that counts, and that considerations in relation to reverence's rebuttable presumption are less relevant. If an informed and capacitous patient has expressed a voluntary, clear and settled wish to die, it is of course within their reach to end their own life with the involvement of no one else should they wish to do so. English law no longer criminalises suicide. Yet, some patients who have made a decision to die will be physically incapable of taking their own life, for a variety of reasons. In this scenario some type of assistance is required but, as English law currently stands, no lawful help is permissible. Where the involvement of a third party is required to assist another to die, arguably the debate concerning the appropriate role, if any, of the criminal law moves more sharply into focus. It is here where reverence for life, as a framework for locating the role of the criminal law, carries with it certain advantages. Before we elaborate on these issues, we first consider how the law has recently dealt with cases involving active assistance.

***Nicklinson***

The high‐profile case of *Nicklinson* primarily concerned the ban on encouraging or assisting suicide under the Suicide Act 1961. The main appeal converged on the ongoing development and possible expansion of the right of autonomy under Art 8 of the European Convention on Human Rights. 82
See *Nicklinson*, *Pretty and Purdy*, above n 8.
 The appellants failed in their attempt to persuade the majority of the Supreme Court that the blanket ban on assisting a suicide was incompatible with Art 8 (1). However, in addition to those dissenting, 83
Lady Hale and Lord Kerr.
 three Justices occupying what might be regarded as the middle ground in this case suggested that a future attempt might be successful if Parliament failed to consider the ongoing interference with autonomy in end‐of‐life issues, 84
See the judgments of Lord Neuberger, Lord Wilson and Lord Mance.
 which is firmly established under Art 8. 85
See eg *Haas* *v* *Switzerland* (2011) 53 EHRR 33; *Koch* *v* *Germany* (2013) 56 EHRR 6; and *Gross* *v* *Switzerland* (2014) 58 EHRR 7.
 A number of the Justices reflected on the diverse legal and moral arguments that have shaped this debate, including the importance of the value of life.

Lord Neuberger considered various dimensions to the ‘sanctity’ or ‘primacy’ principle, as he put it, and how it relates to the ban on assisted suicide. In relation to the life of a person seeking to die, he argued that the decriminalisation of suicide had substantially undermined the principle, stating: ‘… if the primacy of human life does not prevent a person committing suicide, it is difficult to see why it should prevent that person seeking assistance in committing suicide’. 86

*Nicklinson*, above n 8, at [90].
 This point raises comparative questions of legal and ethical consistency within the laws regulating death and the varied, often conflicting, messages that such laws seem to convey. Concerns over inconsistency and illogical distinctions permeate the end‐of‐life debate and the judgment in *Nicklinson* reflects this. For example, comparing the appellants’ case to other end‐of‐life dilemmas, Lord Neuberger suggested that withdrawing life‐sustaining treatment is a ‘more drastic interference in that person's life and more extreme moral step’ 87
Ibid, at [94].
 than assisting in a suicide where the person wanting to die is clearly in command.

Here, Lord Neuberger neatly illustrates some of the difficulties that are associated with substitute decision making in a situation where the patient lacks volition, as opposed to those cases in which a patient is able to articulate a view. For him, it would seem that having to make a decision that would end the life of a patient when it is not possible to ascertain what that patient's wishes would have been is of greater cause for concern than those cases that rest on the expression of an autonomous wish of a competent patient to receive assistance to die. It follows that reverence for life may be more helpful, at least to Lord Neuberger, in cases where withdrawal of treatment is the main issue, as it may provide some substantial guidance as to when any presumption in favour of preserving life could be reasonably rebutted. Yet, that is not to say that reverence is incapable of providing some useful guidance as to when it may be appropriate for the law to tolerate certain types of assistance in situations where patients have requested it, and we elaborate on this in our principled approach subheading below.

Lady Hale expressed similar concerns when she compared the request made by the late Mr Nicklinson and Paul Lamb to other end‐of‐life decisions, such as Ms B's successful appeal to have life‐sustaining ventilation withdrawn in order to allow her to die. 88
See *B* *v* *NHS Hospital Trust* [2002] EWHC 429 (Fam); [2002] 2 All ER 449.12242880
 Commenting that although the acts/omissions distinction sanctioned by the House of Lords in *Bland* makes some legal sense, Lady Hale observed that it must often make little sense to those affected who are denied a choice. 89

*Nicklinson*, above n 8, at [302]–[304]; and *Bland*, above n 8.



We can thus see that while the various issues that speak to assisted dying and treatment withdrawal cases perhaps raise different issues at first blush, in so far as the value of human life is a central feature in both types of case, they remain irrevocably intertwined. This is especially so when fundamental concerns about safeguarding the vulnerable are considered. To the extent that the objection to legal assisted dying rests on concerns over preserving *other* human lives, Lord Neuberger suggested that it does little more ‘… than replicate concerns about the lives of the weak and vulnerable’. 90
See *Nicklinson*, above n 8. Lord Kerr agreed with Neuberger regarding the logic of using the sanctity argument to prevent those who need assistance while those able to act independently are not prevented [358].
 Lord Neuberger was also influenced by an argument previously used by another assisted dying litigant, Debbie Purdy. 91

*Purdy*, above n 8.
 Agreeing that concerns about legal change based on the sanctity of life can be reversed, he pointed out that evidence shows that ‘some people with a progressive degenerative disease feel themselves forced to end their lives before they would wish to’, 92

*Nicklinson*, above n 8, at [96].
 while still able to act alone. Thus, Lord Neuberger argued, the blanket ban ‘may serve to cut short their lives’. 93
Ibid.
 Similar concerns were evident when Lord Neuberger mentioned the shortcomings of the current approach, which leans against prosecution and offers only retrospective assessment of the victim's circumstances and reasons for wanting to die once it is too late to prevent the suicide. 94
Ibid, at [108].



The importance of the value of life emerged repeatedly and, coincidentally, Lord Sumption spoke of ‘reverence for human life for its own sake’. 95
Ibid, at [209].
 Lady Hale alluded to the religious foundation of the principle, stating that ‘respect for the intrinsic value of all human life is probably the most important value in Judaeo‐Christian morality’. 96
Ibid, at [311].
 This, she suggested, should absolutely justify refusing to oblige a person to help another to commit suicide, but it would not ‘so obviously justify prohibiting those who freely judged that, in the circumstances of a particular case, there was no moral impediment to their assisting suicide’. 97
Ibid.



This view accords with Lady Hale's earlier House of Lords’ judgment in *Purdy*, 98

*Purdy*, above n 8.
 in which she clearly identified the competing interests that collide with maintaining a respect for the intrinsic value of human life above all else. She acknowledged the tension in stating that ‘it is not for society to tell people what to value about their own lives. But it may be justifiable for society to insist that we value their lives even if they do not.’ 99
Ibid, at [68].
 This clear recognition of the value of human life in society was somewhat tempered though by the fact that she suggested elsewhere in her judgment that ‘if we are serious about protecting autonomy we have to accept that autonomous individuals have different views about what makes their lives worth living’. 100
Ibid, at [66].
 This highlights both the tension between these competing ethical concerns and the importance of establishing a compromise between valuing human life and valuing autonomy.

Interestingly, Lady Hale stressed that it was important that some attention was paid to the reasons why a person wished to be helped to end his or her life. 101
Ibid, at [68].
 For her, then, it seems clear that there may be some situations in which preserving life at all costs on the basis that it has an intrinsic value is not a desirable position for the law to adopt. Factors pertaining to *why* the patient may be requesting assistance could be sufficiently convincing so as not to warrant prosecution if such assistance is in fact provided.

Such willingness to open the door to rebutting the presumption in favour of life, for a person whose Art 8 rights are engaged and who wishes to receive assistance in suicide, seems to represent a significant development in judicial thinking. In weighing the presumption for life against other fundamental ethical concerns (mainly autonomy, but also dignity), several members of the Supreme Court have provided guidance for Parliament or even for future cases. While the most recent discussions in the House of Commons indicate that legislative reform is not imminent 102
The Assisted Dying (No. 2) Bill, MP Rob Marris’ private member's bill – which, like the recent HL Bill (below) sought to legalise physician‐assisted suicide (PAS) for terminally ill, mentally competent people expected to die naturally within 6 months – was defeated at second reading on 11 September 2015.
 – primarily, it seems, because of the possible danger for vulnerable people – the amendments to the Assisted Dying Bill 103
House of Lords Bill (24 (2013), 6 (2014)). The Bill reached Committee Stage before parliamentary time elapsed; however, it is significant that some of the debates reflected an appreciation of the judgment in *Nicklinson*.
 in the House of Lords demonstrated that resolving the valid competing concerns is challenging but not impossible. 104
See *Hansard* HL Deb, vol 756, cols 1851–1898, 7 November 2014; and cols 1001–1047, 16 January 2015.
 By offering a more principled approach to rebutting the presumption in favour of preserving life, the reverence principle is capable of addressing such challenges. Thus, in the final section of this paper, we summarise recent developments in relation to treatment withdrawal, before tentatively suggesting a more progressive and consistent approach in assisted dying cases.

## Rebutting the Presumption: Reverence Not Sanctity?

Coherent and ethically acceptable principles have developed in relation to certain questions about life and death. We know conclusively that a competent adult has an inviolable right to refuse life‐sustaining treatment. 105


*B* *v* *NHS Trust* , above n 88.
 This right to die via withdrawal can extend to those who create a legally valid AD, and to those in a vegetative state, despite the argument that removing life‐sustaining sustenance in such circumstances may be regarded as indistinguishable from euthanasia. 106
See eg JK Mason and GT Laurie *Mason and McCall Smith*'*s Law and Medical Ethics* (Oxford: Oxford University Press, 9th edn, 2013) p 549 ; and D Orentlicher ‘The alleged distinction between euthanasia and the withdrawal of life‐sustaining treatment: conceptually incoherent and impossible to maintain’ (2012) 1998 U Ill L Rev 837.12071210
 Rebutting the presumption in favour of life for those in MCS or a similar condition will often hinge on a best interests evaluation, which has been subject to significant judicial discretion. The decision in *Aintree* has confirmed that the test as to what is a worthwhile treatment must be influenced by the patient's wishes, thus finally providing a more satisfactory interpretation of the relevant provision of the Mental Capacity Act 2005. 107
Mental Capacity Act 2005, s 4(6)(a)–(c).



Moreover, questions of the burdens and benefits of treatment, together with assessing the experiences of the patient, unavoidably, and rightly, involve certain quality‐of‐life determinations. Accordingly, the principle of preserving life needs to holistically reflect these wider concerns in a coherent and principled fashion. Each case will be decided on its individual facts but we can, and should, endeavour to have a consistent and ethically sound approach. If the early indicators from the cases discussed above prove to reflect the general post‐*Aintree* approach, we may be satisfied that the Supreme Court in *Aintree* has done enough to sustain a more ethically coherent approach that properly recognises the importance of the patient's subjective preferences and values within a holistic methodology for determining best interests, albeit in the wake of *M v N* there ought to be a degree of caution so as to ensure that it does not swing too far in one direction.

Cases concerning active interventions foreshortening life, however, remain in thrall to sanctity concerns and, understandably, the courts have been anxious to avoid allegations of usurping parliamentary sovereignty. But as Lord Neuberger and Lady Hale indicated in *Nicklinson*, other ethical concerns should be regarded as equally (and sometimes more) important than traditional sanctity considerations in order to facilitate a more subtle approach to this dilemma. In *Bland*, Lord Goff argued that the right to self‐determination should not be eclipsed by the fact of incompetence. 108

*Bland*, above n 8, at 866.
 Similarly, we question whether this right must continue to be eclipsed by the fact of physical inability to end one's own life by active measures. Notwithstanding the importance of protecting the vulnerable, the recognition of the autonomous right of the competent individual to choose death in order to avoid terrible suffering should lead to a more nuanced and sensitive approach from the law. Thus a powerful imperative to develop a more coherent and principled approach regarding the presumption in favour of preserving life emerges.

## Devising a More Principled Approach

The final section of this paper will begin to suggest an approach that accommodates, to an extent, ethical principles such as dignity and autonomy, while protecting the vulnerable and maintaining that life is presumed to be intrinsically valuable according to the reverence principle. Devising a principled approach to rebutting the presumption for life preservation first requires some consideration of what we understand in relation to dignity and autonomy, and in particular the law's relationship with autonomy. This is vital in order to address how reverence for life may be construed as a framework that correlates with the criminal law.

Principles of dignity and autonomy are notoriously ambiguous and subject to diverse and mercurial interpretations; 109
See eg LR Kass ‘Death with dignity and the sanctity of life’ in KoganBS (ed) *A Time to be Born and a Time to Die*: *the Ethics of Choice* (New York: Aldine de Gruyter, 1991) p 117 ; R Macklin ‘Dignity is a useless concept: it means no more than respect for persons or their autonomy’ (2003) 327(7429) Br Med J 1419 1468463310.1136/bmj.327.7429.1419PMC300789; D Schroeder ‘Dignity: Two riddles and four concepts’ (2008) 17(2), Camb Q Healthcare Ethics 230 10.1017/S096318010808026218312738; G Dworkin The Theory and Practice of Autonomy (Cambridge: Cambridge University Press, 1988); O O'Neill Autonomy and Trust in Bioethics (Cambridge: Cambridge University Press, 2002); S McLean *Assisted Dying*: *Reflections on the Need for Law Reform* (London: Routledge, 2007).
 nevertheless, we attempt a necessarily brief exploration in the context of reverence, beginning with dignity.

Mary Neal's work is useful in drawing from the conflicting accounts within the law and academic literature in order to consider the challenges of understanding dignity as a concept or principle, an intrinsic human value, a virtue, a norm and/or a right. 110
See M Neal ‘Respect for human dignity as “substantive basic norm”’ (2014) 10 Int'l J Law in Context 26 ; M Neal ‘Dignity, law and language‐games’ (2012) 25 Int'l J for Semiotics L – Revue Internationale de Sémiotique Juridique 107 ; M Neal ‘“Not gods but animals”: human dignity and vulnerable subjecthood’ (2012) 33 Liverpool L Rev 177.
 Dignity's ‘messy appeal’, according to Neal, is that ‘the idea of dignity attracts us by seeming to chime with some of our ethical intuitions, but as we attempt to analyse it, we come up against an array of possible meanings’. 111
Ibid, Neal ‘Human dignity and vulnerable subjecthood’, at 179.
 Such ambiguity means that it is problematic to attempt to effectively deploy dignity in ethico‐legal dilemmas, but we suggest that there is a meaningful concept of dignity in relation to assisted dying. We agree with Neal that to conflate dignity with autonomy (using Kantian principles) only accounts for a segment of dignity's potential application. Dignity is not essentially synonymous with autonomy, yet, where assisted dying is concerned, the two seem invariably connected.

Where a competent adult expresses an autonomous desire to die rather than to continue to endure what they regard as an undignified and unendurably unpleasant or painful existence, or to avoid what is likely to be – according to their understanding – an undignified dying process, dignity and autonomy seem to be conjoined. The way in which a person values their life, both physically and biographically, is often connected to the way in which that person understands and values their human dignity. This is necessarily nuanced and subjective, yet the narratives behind the important legal challenges (*Pretty*, *Purdy*, *Nicklinson*), 112
Above n 8.
 suggest that people who seek death in this context share a similar perception of the relationship between autonomy and dignity. Indeed, the ‘right to a dignified death’ is the primary campaigning slogan of the main organisation pushing for legal change in the UK. 113
Dignity in Dying, see http://www.dignityindying.org.uk/about‐us/ (accessed 14 February 2016).
 And in jurisdictions where PAS is legal, notions of autonomy and dignity seem crucial to those seeking PAS. 114
See eg S Fischer et al ‘Reasons why people in Switzerland seek assisted suicide: the view of patients and physicians’ (2009) 139 Swiss Med Weekly 333 10.4414/smw.2009.1261419529991; CDM Ruijs et al ‘Unbearable suffering and requests for euthanasia prospectively studied in end‐of‐life cancer patients in primary care’ (2014) 13(1) BMC Palliative Care 62.2558724010.1186/1472-684X-13-62PMC4292985



With respect to autonomy per se, its compatibility with reverence for life is a little more difficult to articulate. This tension is born out of a problem that we made explicit reference to earlier; arguments in favour of relying on reverence for life are more convincing where a patient is in a PVS or MCS and lacks capacity, as opposed to where a patient can express her own wishes. If a patient is capable of competently expressing a clear and settled wish to die, it may reasonably be suggested by some that all other considerations become redundant and that there is no need for recourse to reverence's purported compromise. Effectively, there would be no need to introduce any objective considerations into the equation that may point in the direction of preserving life because autonomy should prevail. With this in mind, it is sometimes difficult to conceive of a compromise between giving effect to autonomy on the one hand, and any objective valuing of life on the other. The arguments are often presented as polar opposites and the accusation could be made that in embracing some objective considerations when assessing the value of life, reverence for life actually privileges preservation of life in a way that is not dissimilar to sanctity and inviolability.

This overlooks certain key features of the suggested role of reverence. First, it fails to recognise that reverence only purports to offer a framework in which the criminal law can be located and, secondly, that viewing reverence as a framework is the very thing that allows it to neatly capture how the law treats and respects certain autonomous decisions. The law respects and permits certain autonomous choices, but there are limits to the extent that it does so; the conception of reverence offered here reflects this, and so fits neatly into the structure of the criminal law. 115
For two interesting decisions that illustrate the extent to which the criminal law permits autonomous choice in relation to certain activities, but not others, see *R* *v* *Wilson* [1997] QB 47; *R* *v* *Brown and Others* [1994] 1 AC 212.
 It does not privilege preservation of life over autonomy, but it does take as its starting point the recognition of the intrinsic value of all life, and its default position is that life should therefore be protected and preserved. However, it also accepts that in certain situations factors will be present that would cause that presumption in favour of preserving life to be rebutted and so would allow certain patients the right to receive assistance to die, and would not penalise that patient or indeed any individual who provided the assistance.

Thus, if a patient made an autonomous request to receive assistance to die, reverence would not automatically support the criminal law countenancing that choice, without certain ‘other’ factors being present that were sufficiently convincing to rebut reverence's presumption in favour of life. If, however, those factors were present, reverence would support the criminal law in respecting the patient's autonomous choice. An autonomous wish, in the context of reverence, could therefore be described as necessary but not sufficient to prevent the criminal law from intervening in respect of every occurrence of assisted suicide.

By way of example, both authors would favour a more permissive law in respect of PAS. However, neither of us would feel comfortable in supporting a situation in which medical assistance could be provided, without any legal ramifications, for example, to an 18‐year‐old patient who sought to end his life simply because he was feeling miserable that he had just been rejected by his girlfriend. His choice may well be autonomous in the sense that *he* has concluded that his life is no longer of value, and he may well have made a settled and informed choice, but reverence would not support his purported autonomous choice as being one that is capable of rebutting the presumption in favour of preserving life. In short, reverence would require something more for the law to give effect to the autonomous wish of the patient.

With this in mind, change the above hypothetical scenario slightly. Our patient is now a 50‐year‐old man who has requested assistance to die because of mounting suffering and (what he sees as) indignity as a result of motor neurone disease, which is an incurable, degenerative disease. In this scenario, his autonomous wish, coupled with the objective factors that are present as a result of his condition, may bring him within the reach of reverence supporting a choice to receive assistance to die, provided that those objective factors were deemed sufficient to rebut the presumption in support of maintaining life.

We now begin to see that reverence could equally have a role in active assistance cases as well as in withdrawal cases by functioning within a system of law that respects certain choices made by individuals, but not every choice, especially where there may be a strong justification for impinging on that choice. It is in this respect that considering certain objective factors in any assessment of the value of life could usefully operate as an appropriate counterbalance to safeguard against exploitation of the vulnerable, to prevent instances of assisted suicide from becoming too commonplace in society and thus spiralling out of control, and to not diminish completely the importance of recognising, at least to an extent, the intrinsic value of life. A corollary of this conception of reverence is that, in the same way that some objective determinations are needed to weigh up where the presumption in favour of preserving life could be rebutted in MCS/PVS cases, so too does some emphasis need to be placed on objective factors in active assistance cases. In the context of active assistance, identifying precisely what objective factors ought to be taken into account, and the emphasis that should be placed on them, is certainly problematic. Thus, it becomes essential to at least make some attempt to define what may amount to appropriate criteria that may be capable of rebutting any presumption for life.

Needless to say, agreeing the criteria for eligibility for an assisted death is no easy task. If terminal illness expected to prove imminently fatal is the requirement, many people, such as the appellants in *Nicklinson*, will not qualify. Alternatively, if eligibility rests on an assessment of the extent and intractability of suffering, there will be concerns that the lives of the seriously ill and disabled might be seen as less valued and so the system put in place would need to include safeguards to avoid this. Discussion of exactly what this criteria should demand is beyond the scope of this paper, yet we agree with Lady Hale that ‘it would not be beyond the wit of a legal system to devise a process for identifying those people, those few people, who should be allowed help to end their own lives’. 116

*Nicklinson*, above n 8, at [314].
 Lady Hale suggested four essential requirements: capacity, no undue influence, full knowledge of their situation (the options available and consequences of the decision) and finally, the person must be unable, due to physical incapacity or frailty, to act alone. 117
Ibid.



Difficulties arise, however, where one of the qualifying factors would rest on the patient being physically incapable of acting alone. This requirement is problematic for two reasons. First, if this factor became a prerequisite for the non‐prosecution of anyone who provided assistance, it might result in many patients having to postpone a decision to end their life until the point at which they could not actually act themselves. For some diseases, such as cancer, this point might not come until the closing weeks or days of life, which might subject the patient to prolonged pain and suffering. This may be an unintended, and indeed undesirable, consequence of including this particular qualifying criterion. As it stands, some patients seek to avoid this very situation by requesting assistance to end their own lives at an earlier point, before they become completely dependent on others, so that they can avoid the discomfort, indignity and trauma that will occur in the latter stages of certain illnesses. It follows that some patients will simply ‘desire’ assisted suicide at an early stage in proceedings, rather than actually ‘requiring’ it at a point at which they can no longer act themselves. Insisting that a patient has to be unable to act alone before the law will allow assistance would therefore be problematic. Many patients who simply desire an earlier dignified death, and who seek the help of someone to facilitate this either by travelling with them, or by making arrangements for them in some other way, as frequently happens at present, would fall outside the scope of the qualifying criteria and thus would not trigger reverence's rebuttable presumption. This could well have the effect of causing those who have provided the assistance to be prosecuted more frequently than is currently the case.

A second concern is that this requirement suggests that those able to act alone can take matters into their own hands by committing suicide unaided. Given that taking one's own life can be difficult, with a risk of botched attempts or resorting to violent means of dying and additional distress for all involved, we see a further justification for avoiding the requirement of physical inability to act alone.

In the light of the above, we tentatively suggest that Lady Hale's final requirement be replaced with the need for a recognised medical condition that is permanent, irreversible and/or degenerative, and that has the potential to cause the patient pain, suffering and indignity if their life is prolonged unreasonably. The existence of such a condition would need to be certified by two medical professionals. This would avoid treating those with a physical disability differently, while also ensuring that the person has legitimate medical grounds to justify assistance. Once determined and assessed, these conditions should be capable of rebutting the presumption for life preservation. Accordingly, if a patient satisfied the objective criteria that we would stipulate, and we acknowledge that the precise contours of this would need to be debated in greater depth, then any compassionate assistance provided by a third party to help a patient travel abroad to receive an assisted death should not be prosecuted. Alternatively, if legislation were enacted to introduce a regulated yet permissive medicalised model of PAS, which in the current climate seems unlikely, then patients who meet the criteria should be entitled to receive medical assistance to die, and any doctor who has operated within the provisions of the legislation should be immune from prosecution for complying with that request.

Rebutting the presumption for life in order to allow any overt assisted dying will, of course, raise many objections, not least from within the medical profession, who, collectively, have traditionally opposed any legal change that might be seen to erode the ethic of ‘caring not killing’. 118
See eg the British Medical Association's Policy on assisted dying, available at http://bma.org.uk/practical‐support‐at‐work/ethics/bma‐policy‐assisted‐dying (accessed 14 February 2016), and Assisted Dying for the Terminally Ill Committee *Assisted Dying for the Terminally Ill Bill tteeics*/*bma‐po* (2005) at 42.
 Accordingly, the autonomy of those called upon to provide assistance must be taken into account. Any lawful assisted dying should recognise a professional right to conscientiously (and sensitively) exempt oneself from participating in PAS, 119
For a discussion, see R Huxtable and A Mullock ‘Voices of discontent? Conscience, compromise and assisted dying’ (2015) 23 Med L Rev 242.10.1093/medlaw/fwv00825957300
 although this right should not allow the medical professionals to abandon patients seeking PAS. It is also important to note that professional medical cooperation and acceptance of PAS will assist in creating the best procedures to ensure that patients are properly cared for within a system that succeeds in safeguarding the vulnerable. Evidence indicates that physical pain is often the most important reason for seeking death, 120
Fischer et al, above n 114, at 333; Ruijs et al, above n 113.
 thus professional involvement will be essential to ensure that people seeking PAS are aware of all the facts, especially other options to alleviate suffering.

Having suggested that greater recognition of dignity and autonomy together with carefully constructed criteria and a process for rebutting the presumption for life could pave the way for a more coherent, consistent and ethical approach, we have left a number of questions unanswered. Some of these include, inter alia, defining precisely when and why the presumption should be rebutted in differing scenarios. In the above discussion, we have offered some examples of where we think it may have been appropriate for this to happen, but they are by no means intended to be an exhaustive list. The principal advantage of adopting reverence would be its fluid, fact‐sensitive nature and so opinions and beliefs as to when and why the presumption should be rebutted will naturally vary. Therefore, as a body of case‐law builds up, it would be of paramount importance to analyse the type of factual scenarios that would warrant considering a departure from the presumption in favour of life and the amount and type of evidence that would be needed in order for the rebuttal to actually bite.

A number of inextricably linked further points flow from this. How would the framework of reverence provide adequate protection for the vulnerable individuals in society? Given that this has proven to be the dominant concern for English law in the end‐of‐life debate, some serious thought would need to be given as to how, if indeed it all, it would be possible to placate those who viewed this as a source of anxiety. Moreover, there is the time‐honoured argument of the slippery slope that is bound to rear its head in opposition. Critics will no doubt point out that if judges are too quick to rebut the presumption, then the importance of *presumption* itself may be eroded and this would do untold damage to recognising the intrinsic value of life, and in turn the law's main priority of preserving life. Clearly, then, there would need to be some guidance as to the exercise, and limits, of judicial flexibility in interpreting the concept of reverence.

These questions are, however, impossible to determine at present, for to answer them would require reverence to be adopted in contemporary legal parlance. This in itself raises a key concern of how judges could be encouraged to incorporate reverence for life and to inject it into mainstream judicial reasoning in a way that does more than simply portray it as yet another mere variance in phraseology, by presenting it, and using it, in the principled way that has been suggested.

*If* those questions can be answered to provide a model that adequately safeguards the vulnerable while maintaining a strong presumption in favour of life, a more coherent, consistent, ethical and secular approach may be identified. Thus reverence could provide an ethically sound and coherent compromise between the argument that life is intrinsically sacred or inviolable and the quality‐of‐life position.

## Conclusion

We sit on a ‘perilous perch’ regarding the endless battles about the intrinsic value of life. 121
Brazier and Ost, above n 4, p 83.
 Religious doctrines enshrining the ‘sanctity’ of life invoke connotations that do not reflect the concerns of a pluralistic and predominantly secular society. Furthermore, as Price argued, sanctity may be a ‘hazardous distraction’, and ‘[R]espect for specific patients not the sanctity of life is the proper legal and ethical guide’. 122


D Price ‘My view of the sanctity of life: a rebuttal of John Keown's critique’ (2007) 27 Legal Stud 549.
 While the concept of sanctity has evolved to encompass the alternative principle of inviolability, this too provides a limited and theoretically flawed approach to this dilemma. Inviolability purports to cast off religious baggage yet causes confusion over possible interpretations of treatment and futility and suffers from too heavy a reliance on double effect, which may be seen as a ‘sop’, rather than a sound way to limit any tendency towards vitalism. Both concepts offer an incoherent and sometimes highly discretionary approach that has generally encouraged an overly paternalistic attitude. Searching for an alternative, ‘respect’ for life seems inadequate for a strong presumption in favour of life. Lord Neuberger's reference to ‘primacy’ might be useful in providing a simple numerical expression conveying importance while avoiding being value‐laden, yet we question whether a term so devoid of ethical significance is desirable. Does the concept of reverence for life assist us, then, in finding a principled compromise and a more coherent approach?

Inevitably, there may be cynicism about the addition of yet another ambiguous term into the never‐ending debate concerning the intrinsic value of human life. There is also the pragmatic question of whether in fact reverence would change anything at all in terms of outcomes in these hard cases. We have suggested that, if incorporated, it may well have the effect of altering the outcome in certain cases. While we would both agree that significant inroads were made by judges in the end‐of‐life cases such as *Bland*, *Purdy* and *Aintree* (at least in the Court of Appeal and the Supreme Court), we would argue that in other contemporary cases such as *W v M and others*, *Aintree* in the Court of Protection, and even *Nicklinson* in the Supreme Court, the use of reverence for life may have helped to hone the restrictive nature of some of the judicial reasoning. Reverence, as we conceive it, may have helped to focus the minds of certain judges so that they were more acutely aware of the fact if the evidence was compelling enough, they were entitled to become unshackled from the narrow view that the law should be predominantly concerned with preserving life at all costs because the intrinsic value of life is sacrosanct. As more and more difficult cases come before the courts concerning MCS patients or, indeed, those suffering from terminal, degenerative conditions who request active assistance to die, the embedded notion of a rebuttal presumption may have a direct bearing by leading to a more compassionate and humane outcome in certain situations. The recent defeat of Rob Marris’ Assisted Dying Bill in the House of Commons indicates that the courts will again be called upon to resolve the tension between the rather nebulous autonomous rights protected by Art 8 ECHR and the need to preserve life for vulnerable people. Moreover, as the law seems to be developing incrementally to give greater respect to the right to autonomy in the medical context, 123


*Montgomery* *v* *Lanarkshire Health Board* [2015] UKSC 11; [2015] AC 1430.
 a patient‐centric approach that includes certain objective considerations, which can be justified in order to protect the vulnerable, seems appropriate.

Accordingly, we are of the view that Brazier and Ost mean reverence for life to be something more than just a linguistic alternative. The idea has a much greater potential in so far as it conveys importance to humanity, offering a less absolute position that reflects the rebuttable presumption for preserving life in a way that might be developed as a coherent compromise between sanctity, inviolability and concerns about autonomy, dignity and the quality of life.

